# Vitamin D and Pigmented Skin

**DOI:** 10.3390/nu14020325

**Published:** 2022-01-13

**Authors:** Carsten Carlberg

**Affiliations:** School of Medicine, Institute of Biomedicine, University of Eastern Finland, FI-70211 Kuopio, Finland; carsten.carlberg@uef.fi

The default supply of vitamin D_3_ to humans is its endogenous production in UV-B-exposed skin [1]. However, changes in lifestyle such as predominant indoor activities combined with textile coverage outdoors necessitate the uptake of this pre-hormone by fatty fish or supplemented dietary products, such as milk and margarine, or direct supplementation via pills [2]. Insufficient vitamin D_3_ production or supplementation causes vitamin D deficiency, which in the long term can lead to bone malformations, such as those observed in rickets [3]. In addition, an insufficient vitamin D status (determined as a 25-hydroxyvitamin D_3_ (25(OH)D_3_) serum level below 50 nM (20 ng/mL)) may cause a malfunctional immune system [4], which manifests as an increased risk for severe consequences of infectious diseases such as tuberculosis [5] or COVID-19 (coronavirus) [6], as well as for the onset and progression of autoimmune diseases such as multiple sclerosis [7] and type 1 diabetes [8].

One key paper published in 2021 in *Nutrients* [9] discusses whether the 15–20 times higher prevalence of vitamin D deficiency in African Americans compared to European-decent Americans contributes to health disparities. They conclude that there is moderate-to-strong evidence that sufficient vitamin D supplementation would reduce the risk not only of rickets, COVID-19, and multiple sclerosis, but also the all-cause mortality rate, adverse pregnancy and birth outcomes, cancer, diabetes, Alzheimer’s disease and dementia, acute respiratory tract infections, asthma exacerbations, and osteomalacia. This long list of diseases may also reflect the on average lower access to health care of African Americans. Nevertheless, it is a strong conclusion that suggests immediate actions for efficient vitamin D_3_ supplementation, which would be a first step towards the better medical service of minorities. This applies not only in the US but to all countries at higher latitudes that for historical reasons have a large number of dark-skinned inhabitants, such as Canada, the UK, and France. Moreover, the south–north migration of more intensively pigmented people arriving in significant numbers in most European countries urges health care authorities to organize their vitamin D_3_ supplementation.

Let us take this question to an evolutionary and molecular perspective [10]. It can be assumed that early human species, which evolved in equatorial Africa, had, like today’s primates, light skin below their dark fur. These hominins lost most of their body hair some 2 million years ago [11], in order to enhance their physical performance via more efficient heat dissipation [12]. Consequently, their skin was selected by evolution for more intensive pigmentation in order to prevent skin cancer and other harm caused by intensive exposure to equatorial sun [13,14]. Our own species, *homo sapiens*, evolved some 300,000 years ago in East Africa; members were rapidly distributed over the whole continent and obviously also had dark skin. The pigmentation of skin and hair depends on the content of the brown–black molecule eumelanin, which is produced in melanosomes from the amino acid tyrosine [15]. Melanosomes are specialized organelles within melanocytes that are transferred to keratinocytes and hair follicles in order to pigment them [16]. The dark pigmentation of the skin is primarily based on eumelanin located in the basal layer of the epidermis [14]. Interestingly, this does not significantly inhibit vitamin D_3_ synthesis, which predominantly occurs in upper skin layers [17,18,19]. For example, traditionally living members of the Tanzanian Hadza population, which expose most of their skin to intensive sun, have an average 25(OH)D_3_ serum level of 110 nM [20].

Some 75,000 years ago, *homo sapiens* started to migrate “out of Africa” to Asia and from there to Oceania and Europe, reaching the Americas some 12,000 years ago [21,22]. Those human populations that continued to live in equatorial regions in Africa, Southern India, and Australia, or in higher altitudes such as the Andes kept their intensive skin pigmentation in order to be protected against photodamage [23]. Recent archeogenomic data suggest that even most Western Eurasian populations had dark skin until some 5–10,000 years ago, although *homo sapiens* arrived there 40,000 years ago. However, variants of the genes *SLC24A5* (solute carrier family 24 member 5), *SLC45A2*, and *OCA2* (OCA2 melanosomal transmembrane protein) [22,24], which encode for proteins functioning as potassium-dependent sodium/calcium exchangers, ion transporters, and pH regulators in melanosomes, respectively [15], spread among some populations in the Near East and Western Asia and led to a reduced production of eumelanin and melanosomes—i.e., to lighter skin. The migration of these populations to Europe and other regions in Asia and their admixture with the native populations lead to the prominent light skin phenotype of many populations in the Northern hemisphere. Nevertheless, the genetic drift in skin color took many dozens of generations and hundreds to thousands of years.

In conclusion, for the longest time of their existence, the members of our species had dark skin combined with a traditional lifestyle that included sufficient exposure to the sun, meaning that sufficient vitamin D_3_ was produced endogenously. Even outside of Africa, migration occurred rather slowly and allowed enough time for the species to genetically adapt to the new environment, which featured less sun exposure. However, the unvoluntary transfer of millions of people from Africa to the Americas during the 16th, 17th, and 18th centuries occurred too fast for them to adjust to the higher latitude and reduced sun exposure. In addition, the industrial revolution of the past 200 years and the changes in the globalized world in the past few decades have created a drastic lifestyle change, with those whose physiology was not adapted to the environment suffering most. All nearly 8 billion of us need to take care regarding our vitamin D status, but for those with dark skin this must be a high priority.
